# Loss of Vascular Endothelial Glutaminase Inhibits Tumor Growth and Metastasis, and Increases Sensitivity to Chemotherapy

**DOI:** 10.1158/2767-9764.CRC-22-0048

**Published:** 2022-07-21

**Authors:** Verra M. Ngwa, Deanna N. Edwards, Yoonha Hwang, Breelyn Karno, Xiaoyong Wang, Chi Yan, Ann Richmond, Dana M. Brantley-Sieders, Jin Chen

**Affiliations:** 1 Program in Cancer Biology, Vanderbilt University, Nashville, Tennessee.; 2 Department of Medicine, Division of Rheumatology, Vanderbilt University Medical Center, Nashville, Tennessee.; 3 Department of Veterans Affairs, Tennessee Valley Healthcare System, Nashville, Tennessee.; 4 Department of Pharmacology, Vanderbilt University School of Medicine, Nashville, Tennessee.; 5 Vanderbilt Ingram Cancer Center, Vanderbilt University Medical Center, Nashville, Tennessee.; 6 Department of Cell and Developmental Biology, Vanderbilt University, Nashville, Tennessee.

## Abstract

Glutamine is the most abundant nonessential amino acid in blood stream; yet its concentration in tumor interstitium is markedly lower than that in the serum, reflecting the huge demand of various cell types in tumor microenvironment for glutamine. While many studies have investigated glutamine metabolism in tumor epithelium and infiltrating immune cells, the role of glutamine metabolism in tumor blood vessels remains unknown. Here, we report that inducible genetic deletion of glutaminase (GLS) specifically in host endothelium, GLS^ECKO^, impairs tumor growth and metastatic dissemination *in vivo*. Loss of GLS decreased tumor microvascular density, increased perivascular support cell coverage, improved perfusion, and reduced hypoxia in mammary tumors. Importantly, chemotherapeutic drug delivery and therapeutic efficacy were improved in tumor-bearing GLS^ECKO^ hosts or in combination with GLS inhibitor, CB-839. Mechanistically, loss of GLS in tumor endothelium resulted in decreased leptin levels, and exogenous recombinant leptin rescued tumor growth defects in GLS^ECKO^ mice. Together, these data demonstrate that inhibition of endothelial glutamine metabolism normalizes tumor vessels, reducing tumor growth and metastatic spread, improving perfusion, reducing hypoxia, and enhancing chemotherapeutic delivery. Thus, targeting glutamine metabolism in host vasculature may improve clinical outcome in patients with solid tumors.

**Significance:**

This study demonstrates a crucial role for glutamine metabolism in tumor endothelium, which may be exploited therapeutically to induce vascular normalization and improve drug delivery in solid tumors.

## Introduction

The vasculature of a solid tumor is characterized by tortuous, leaky, and chaotic networks of irregular endothelial cells (EC) and reduction in perivascular support cells. Through impaired nutrient delivery and increased hypoxia, this dysfunctional network promotes tumor metastasis and resistance to therapy (1–5). Like cancer cells, tumor vascular ECs are highly proliferative and require nutrients to support their rapid growth. Glucose metabolism has been shown to be critical in tumor ECs (5, 6). Targeting PFKFB3 to inhibit endothelial glycolysis promoted tumor vessel normalization, increased drug delivery and decreased metastasis (6). In addition to glycolysis, normal ECs also depend on glutamine metabolism for proliferation and migration (7, 8); however, the role of glutaminolysis in tumor endothelium remains to be determined. This is particularly important, as inhibitors of glutamine metabolism that potentially impact multiple cell types in the tumor microenvironment are in various stages of clinical trials.

Glutamine provides nitrogen for biosynthesis of nucleotides and amino acids, serves as a mitochondrial substrate, and fuels lipid biogenesis (9). Cells readily convert glutamine to glutamate by glutaminase 1 and 2 (GLS1/GLS2), the rate-limiting step of glutaminolysis. High expression of *GLS1* (GLS) is associated with poor prognosis in human cancers (10, 11). In addition, glutamate, a metabolite of GLS, is a precursor of glutathione, a major cellular antioxidant. Glutamine is also the source of α-ketoglutarate, which serves as a substrate for dioxygenases that modify proteins and DNA for epigenetic regulation (12). While the role of glutamine metabolism has been defined for tumor cells and a number of immune cells (13–15), little is known about glutamine metabolism in tumor blood vessels. Here, we assessed if targeting GLS in vascular ECs affects pathologic angiogenesis, tumor blood vessel structure and function, and tumorigenesis and metastasis, as GLS2 is expressed at low level in ECs (16).

To understand the role of vascular endothelial GLS in tumor vasculature, we utilized an inducible transgenic mouse model to delete GLS specifically in the endothelium (GLS^ECKO^) using an inducible endothelial-specific Cre. We showed that vascular endothelial GLS is required for tumor angiogenesis, tumor growth, and metastasis. Our data reveal that GLS loss in endothelium decreases angiogenic sprouting while promoting tumor vessel normalization. GLS^ECKO^ tumors with normalized blood vessels displayed an increase in drug delivery and enhanced antitumor effect of chemotherapy. Mechanistically, we discovered a functional link between GLS and expression of leptin, a key regulator of metabolic homeostasis. Together, these data demonstrate a crucial role for glutamine metabolism in tumor endothelium, which may be exploited therapeutically to induce vascular normalization and improve drug delivery in solid tumors.

## Materials and Methods

### Mouse Models

Animal care and experimental procedures were performed under protocols approved by Vanderbilt University's Institutional Animal Care and Utilization Committee. All mice used in this study were immunocompetent and housed in a nonbarrier animal facility. GLS^fl/fl^ (C57BL/6) mice were generated as described previously (17) and provided by Dr. Jeff Rathmell (Vanderbilt University, Nashville, TN). CDH5-Cre^ER^ mice (C57BL/6) were originally generated in Dr. Ralf Adam's laboratory (Max Planck Institute, Münster, Germany) and provided by Dr. Hong Chen (Boston Children's Hospital, Harvard Medical School, Boston, MA). To delete GLS specifically in the endothelium (GLS^ECKO^), tamoxifen (#B5965, ApexBio) was reconstituted in sunflower seed oil (#S5007, Sigma-Aldrich) at 15 mg/mL, and a dose of 2 mg/kg was administered to 6 to 8 weeks old mice by intraperitoneal injection for 5 consecutive days.

### Genotyping

Animals were genotyped for Cre and floxed *GLS* alleles. Ear biopsy samples were digested at 55°C overnight in 100 μL BBK buffer [500 mmol/L KCl, 100 mmol/L Tris-HCL (pH 8.3), 1% Gelatin (catalog no. 7765, Sigma-Aldrich), 0.1 mg/mL Proteinease K (catalog no. 740506, Clonetech), 0.45% IGPEAL, 0.45% Tween-20]. The samples were heated at 105°C for 15 minutes and quickly vortexed and centrifuged at 21,000 × *g* for 1 minute. A total of 1 μL of genomic DNA was added to a 25 μL of PCR reaction mix containing 12.5 μL of oneTag Quick-Load 2× Master Mix (catalog no. M0486L, New England Biolabs), 0.5 μL forward/0.5 μL reverse primers and 10.5 μL molecular grade water. Genotyping primers for amplifying Cre (Forward: ACCTGAAGATGTTCGCGATTATCT; Reverse: ACCGTCAGTACGTGAGATATCTT) and GLS (Forward: TAAGATCTGTGGCTGGTCTTCCAGG; Reverse: ACAATGTACCTGAGGGAGTTGACAGG) were purchased from Integrated DNA Technologies.

### Cell Lines and Cell Culture

Mouse mammary cancer cell line E0771 was provided by Dr. Barbara Fingleton (Vanderbilt University, Nashville, TN) and cultured in DMEM (Corning #10-013-CV) supplemented with 10% FBS and 100 U/ml penicillin and streptomycin. Mouse mammary tumor virus–driven polyoma virus middle T antigen (MMTV-PyMT) cells were first isolated (18) and provided by Dr. Rebecca Cook (Vanderbilt University, Nashville, TN) and were cultured in the same condition as above. All tumor cells were maintained at low passages after thaw and cells identities were confirmed by morphology, growth rate, cell signaling, in comparison with phenotypes described in the literature (15, 19). *Mycoplasma* testing was performed on cultured cells every 6 months, most recently in March 2022, using the MycoStrip-*Mycoplasma* Detection kit (rep-mys-10, Invitrogen).

### ECs Isolation and Culture From Tumor-bearing Mice

Murine pulmonary microvascular ECs were isolated from 1 to 3 months old mice after tamoxifen treatment to assess GLS deletion in the endothelium. In some experiments, ECs was isolated in tumor-bearing wild-type (WT; GLS floxed mice) by perfusing the lungs with PBS and trypsin. The cells were cultured for 3–4 days in complete EGM-2 medium containing 100 U/ml penicillin and streptomycin and seeded on 0.1% gelatin-precoated culture plates. The cells were washed with PBS and passage once in complete medium before transducing with Ad-CMV-iCre (catalog no. 1045) or Ad-CMV-control (Ad-CMV-null, #1300; Vector BioLabs) for 24 hours in 0.2% serum EBM-2 medium.

### Tumor Model and Metastasis

For orthotopic models, E0771 cells (2.5 × 10^5^) or MMTV-PyMT (5 × 10^5^) in 1:1 Matrigel/medium were implanted into the #4 inguinal mammary fat pads of 6 to 8 weeks old female GLS^fl/fl^ (WT) and CDH5-Cre^ER^/GLS^fl/fl^ treated with tamoxifen (GLS^ECKO^). Starting on day 7, primary tumors were monitored by measuring the length (*L*) and width (*W*) every other day using a digital caliper. Tumor volume (*V*) was calculated using the formula (*V* = *L* × *W*^2^ × 0.5).

For metastasis studies, E0771 cells were implanted into WT and GLS^ECKO^ mice as described above. Lungs from WT and GLS^ECKO^ were harvested on day 21 and fixed in 10% neutral buffered formalin before hematoxylin and eosin (H&E) staining. Five sections per lung were cut at 100 μm apart and metastases numbers were analyzed in a rasterized manner on 10× magnification fields. To examine lung nodules, E0771 cells implanted in WT and GLS^ECKO^ mice were resected on day 14 after implantation and the lungs harvested on day 20 postresection after perfusing the lungs with India ink (15% India ink, 85% dH_2_O, three drops NH_4_OH/100mL, SKU # STIIN25, Statlab). India ink–injected lungs were washed in tap water and placed in fresh Fekete's solution (95% ethanol, 37% formaldehyde, glacial acetic acid) overnight (20). White tumor nodules against black lung background were counted in a blind fashion.

### Immunofluorescence

Tumor cryosections were prepared as described previously (26). Tumor samples were immediately frozen in OCT compound (#50-363-579, Thermo Fisher Scientific) and kept at −80 degrees until they were further processed. A total of 6–10 μm sections were cut on Leica Cryostat CM1950. Cryosections were fixed in cold acetone for 10 minutes at room temperature followed by two washes with PBS. The sections were incubated in 3% H_2_O_2_ diluted in methanol for 10 minutes at room temperature followed by two washes with PBS 5 minutes each. Samples were blocked with 2.5% goat serum (#G9023, Sigma-Aldrich) in PBS for 1 hour at room temperature. The following antibodies were used for immunofluorescence on cryosections: CD31 (1:100, #102501, BioLegend), alpha smooth muscle actin (α-SMA; 1:100, #M085129-2, Dako), NG2 (1:100, #AB5320, Sigma-Aldrich), and GLS (1:100, #12855-1-AP, Proteintech). Apoptosis or proliferation were assessed by incubating samples with antibodies against cleaved caspase-3 (1:100, #9664, Cell Signaling Technology) or Ki-67 (1:100, #14-5698-80, eBioscience), respectively. All primary antibodies were incubated overnight at 4 degrees, followed by secondary antibodies for 1 hour at room temperature. Secondary antibodies used were goat-anti-rat–Alexa Fluor 594 (#A-11007; Invitrogen), goat-anti-mouse–Alexa Fluor 488 (#A-11001; Invitrogen), goat-anti-rabbit-Alexa Fluor 488 (#A-11006, Invitrogen) and goat-anti rabbit-Alexa Fluor 594 (#A-11012, Invitrogen). Unless indicated, all secondary antibodies were used at 1:500 dilution. The α-SMA staining was performed using mouse on mouse Elite Peroxidase kit (#PK-2200, M.O.M, Vector Laboratories) to reduce background. Tumor sections were mounted using SlowFade Diamond antifade reagent containing DAPI (#S36963, Molecular Probes). Images were taken by an Olympus inverted fluorescence microscope and processed by using the Cellsens Dimension software program. Six to 10 random fields (10× or 20× magnification) were taken per tumor section and analyzed using the NIH Image J software.

### Tumor Hypoxia and Blood Vessel Perfusion Assays

To assess tumor hypoxia, E0771 cells were implanted into WT and GLS^ECKO^ mice as described above. On day 21 after implantation, hyproxyprobe 100 μL (14 ng/mL, Item# HP2-100Kit) was injected intravenously for 2 hours before tumors were harvested. CD31 staining on cryosections was performed as described above. Hypoxia region was determined by assessing pimonidazole-positive area per field in each tumor sample using Image J software. For vessel perfusion analysis, E0771 tumor-bearing mice were injected intravenously with 100 μL of Tomato Lectin (#DL-1174, Vector Laboratories) 10 minutes before tumors were harvested and processed for CD31 staining as described above. The perfused area was defined as a percentage of Lectin^+^CD31^+^ of the total CD31^+^ area using Image J. Five to 10 random fields (20× magnification) were taken per tumor section and analyzed using the NIH Image J software.

### Treatment of E0771 Tumors with Chemotherapeutic Agents and GLS inhibitor

To examine the effect of vascular GLS deletion in response to chemotherapy, E0771 cells were implanted into WT and GLS^ECKO^ mice as described above. On day 21 after implantation, tumor-bearing mice were treated with Doxorubicin HCl (5 mg/kg, NDC 63323-883-05, Fresenius Kabi) via retro-orbital injection (intravenous). Tumors were harvested 10 minutes later. Doxorubicin (DOXO) is autofluorescent and was analyzed in conjunction with CD31 as described above to examine tumor drug delivery. To evaluate antitumor effect in a separate cohort of animals, tumor-bearing mice received cisplatin (Cpt) (4 mg/kg, intraperitoneal injection) every other day for a total of seven doses starting on day 11. Lungs from these mice were perfused and fixed in 10% neutral buffer formalin and sections stained with H&E to analyze lung metastasis. Five sections per lung were cut at 100 μm apart. To inhibit GLS pharmacologically, a separate cohort of WT female C57BL6 mice at age 7 weeks were inoculated with E0771 (2.5 × 10^5^ cells). The mice were randomized on day 7 and treated with either vehicle or CB-839 (50 mg/kg, #HY-12248, MedChemExpress) by intraperitoneal injection before treating with Cpt (4 mg/kg) or PBS starting on day 11. Tumor volumes were measured with a digital caliper and calculated as length × width^2^ × 0.5.

### Leptin Treatment of E0771 Tumor-bearing Mice

To examine the effect of vascular GLS deletion in response to exogeneous leptin treatment, E0771 cells were implanted into WT and GLS^ECKO^ mice as described above. On day 9 after tumor inoculation, mice were randomized and treated daily with either PBS or recombinant leptin 1 mg/kg (L3772-1MG, Sigma-Aldrich) through intraperitoneal injection until day 24 before the mice were sacrificed on day 25. Tumor volume change was calculated as [(*V*_final_ − *V*_initial_)/*V*_initial_]: where *V*_final_ = volume on last day of treatment; *V*_initial_ = volume on first day of treatment. Tumor sections were cryosectioned to examine CD31 costained with α-SMA. Five to 10 random fields (20× magnification) were taken per tumor section and analyzed using the NIH Image J software.

### Cytokine Array

Cytokine antibody array analysis was performed on tumor lysates from WT (*n* = 2) and GLS^ECKO^ (*n* = 2) using the Mouse Cytokine Array G3 (catalog no.: AAM-Cyt-G3-4, RayBiotech) according to manufacturer's protocol. Brief, tumors were homogenized in 1 × lysis buffer and centrifuged to collect supernatants. A total of 100 μg of proteins from each sample were analyzed. Fluorescence quantification of glass microarrays was obtained by a laser scanner (GenePix4000B; Axon Instruments). All raw cytokine array intensity data were normalized to the mean intensity of the WT according to the instructions. Each sample was analyzed as duplicate.

### Western Blot Analysis

For immunoblotting, precleared lysates were electrophoresed by SDS-PAGE and transferred to nitrocellulose membranes, which were blocked for 1 hour in 5% nonfat dry milk. Membranes were incubated with GLS primary antibody dilution at 1:1,000 (#12855-1-AP, Proteintech) overnight, followed by incubation with secondary antibody for 1 hour at room temperature, and imaged using LI-COR Odyssey. Measured proteins were normalized using tubulin as a control.

### ELISA

Whole tumor lysates from WT and GLS^ECKO^ were homogenized under cold conditions. The homogenate was centrifuge for 10 minutes at 15,000 × *g* at 4°C, and the supernatant was collected. The supernatant was directly used for leptin quantification using the mouse/rat leptin ELISA kit (#MOB00B, R&D Systems), according to the manufacturer's instructions.

### Glutamate Assay

Intracellular glutamate concentrations were determined in duplicate using the glutamate assay kit (MAK004-1KT, Sigma-Aldrich) according to the manufacturer's instructions and as described previously (22). ECs isolated from GLS^fl/fl^ mice were treated with Ad-CMV-control (WT) or Ad-CMV-iCre (GLS^ECKO^) as described above in 0.2% serum EBM-2 medium. The cells were fed with complete medium after 24 hours for an additional 24 hours. ECs were then starved from glutamine for 24 hours and were stimulated with l-glutamine (2.5 mmol/L) in EBM-2 medium supplemented with 0.2% FBS for 30 minutes. Glutamate concentrations were calculated from known standards, and all data are normalized on the basis of baseline values.

### qRT-PCR Assay

Total RNA was isolated and reversely transcribed using the RNeasy kit (Qiagen, #74104) and iScript, respectively, according to the manufacturer's instructions. qPCR was performed using the SYBR Green PCR Master Mix (#4368706, Thermo Fisher Scientific) on StepOne system (Applied Biosystems). Each condition was assayed in triplicate. Relative quantification was obtained by comparative *C*_t_ method and relative mRNA expression was normalized to Acta2 housekeeping gene. The primer sequences used for each gene are as follows: mouse *gls*, forward: 5′- GTACAGTCTCTGTGGCTTGG -3′ and reverse: 5′- CAGTTAGCGGCTCATTCAC -3′; mouse *leptin*, forward: 5′- TGCTGCAGATAGCCAATGAC-3′ and reverse: 5′-AGTAGAGTGAGGCTTCCAGGA-3′; mouse leptin receptor, forward: 5′-ACACTGTTAATTTCACACCAGAG-3′; and reverse: 5′-TGGATAAACCCTTGCTCTTCA-3′; *acta2,* forward: 5′-TGACGCTGAAGTATCCGATAGA-3; reverse: 5′-GTACGTCCAGAGGCATAGAGG-3′. Quantitation was performed using the ΔΔ*C*_t_ method.

### Statistical Analysis

Prism software was used to statistically analyze the results. Student *t* test was used to compare two experimental groups. One or two-way ANOVA were used when comparing multiple experimental groups simultaneously: a significant ANOVA test shows that there was a significant difference among the groups and was corrected with either a Tukey, Dunnett, or Sidak multiple comparison test. Error bars represent SEM and alpha was set at 5%.

### Data Availability Statement

RNAseq raw data for this study were generated at BGI Americas. Derived data supporting the findings of this study are available in the Supplementary Data. For additional materials and methods, see the Supplementary Materials and Methods including tables.

## Results

### Loss of Vascular Endothelial GLS Reduces Breast Cancer Growth and Metastasis

**FIGURE 1:**
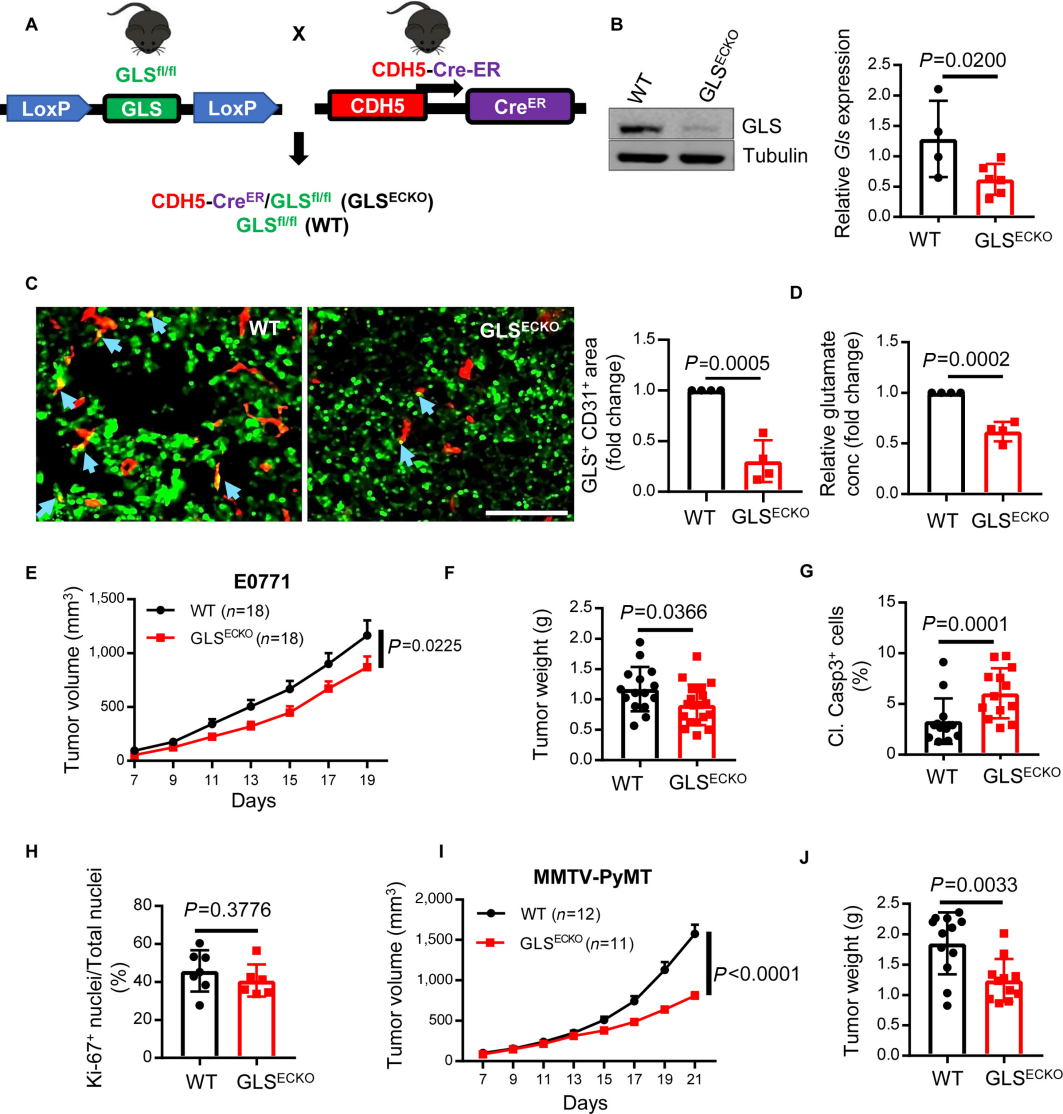
Loss of vascular endothelial GLS reduces breast cancer growth. **A,** A schematic for inducible EC-specific GLS knockout model (GLS^ECKO^). **B,** Western analysis and qRT-PCR of mouse lung microvascular ECs showing GLS loss in GLS^ECKO^ samples. **C,** Dual immunofluorescence images and quantification showing GLS (green) in CD31^+^ (red) blood vessels of E0771 tumors grown in WT versus GLS^ECKO^ mice (*n* = 4 mice per group; Scale bar: 200 μm). Light blue arrows, GLS^+^ tumor ECs. **D,** Intracellular glutamate concentration measured from lung microvascular ECs isolated from GLS f/f mice and treated with Ad-control (WT) or Ad-Cre (GLS^ECKO^). **E,** E0771 tumor growth curves. *P* = 0.0225, two-way ANOVA with Sidak multiple comparisons correction test. **F,** End-stage E0771 tumor weight in WT and GLS^ECKO^ mice (*n* = 15–18). **G,** Cleaved-caspase quantification in WT versus GLS^ECKO^ E0771 tumors (*n* = 10–13 mice per group). **H,** Ki-67 quantification in WT versus GLS^ECKO^ E0771 tumors (*n* = 6–7 mice per group). **I,** MMTV-PyMT tumor growth curves (*n* = 11–12), *P* = 0.0001, two-way ANOVA with Sidak multiple comparisons correction test. **J,** End-stage MMTV-PyMT tumor weight in WT and GLS^ECKO^ mice (*n* = 11–12). All data are presented as mean ± SEM from two or three independent experiments. *P* values of **B**, **C**, **D**, **F**, **G**, **H**, and **J** were determined by two-tailed unpaired Student *t* test.

To investigate the role of GLS in tumor vasculature, we crossed C57BL/6 mice harboring floxed *GLS* alleles (GLS^fl/fl^, referred to as WT) with C57BL/6 mice expressing tamoxifen-inducible Cre recombinase (Cre^ER^) under the control of the *Cdh5*/vascular endothelial-cadherin gene promoter. Following tamoxifen treatment, EC-specific loss of GLS mice (referred to as GLS^ECKO^) were generated (Fig. 1A). GLS^ECKO^ mice were viable and healthy before and following tamoxifen treatment, suggesting that GLS is not required for the survival of vascular ECs in adult animals.

To induce Cre activity and delete endothelial GLS, 6–8 weeks old female mice were injected with tamoxifen daily for 5 consecutive days. E0771 tumor cells or cells derived from the MMTV-PyMT transgenic model (18) were then orthotopically implanted into mammary fat pad of GLS^ECKO^ and WT control mice. Loss of GLS was confirmed by qRT-PCR and Western blot analysis in isolated lung microvascular ECs (Fig. 1B), as well as colocalization of anti-GLS and anti-CD31 immunofluorescence in tumor sections (Fig. 1C). Functionally, GLS deletion reduces intracellular glutamate concentration (Fig. 1D). Endothelial GLS loss resulted in a moderate but consistently significant decrease in primary tumor volume over time (Fig. 1E) and tumor weight at harvest (Fig. 1F). Furthermore, the percentage of cleaved caspase 3–positive cells increased significantly (Fig. 1G; Supplementary Fig. S1A) reflecting apoptosis in GLS^ECKO^ tumors while there was no change in the percentage of Ki-67–positive cells (Fig. 1H; Supplementary Fig. S1B). In the MMTV-PyMT model, we also observed decrease in primary tumor volume and tumor weight when GLS was deleted in the endothelium (Fig. 1I and J).

**FIGURE 2:**
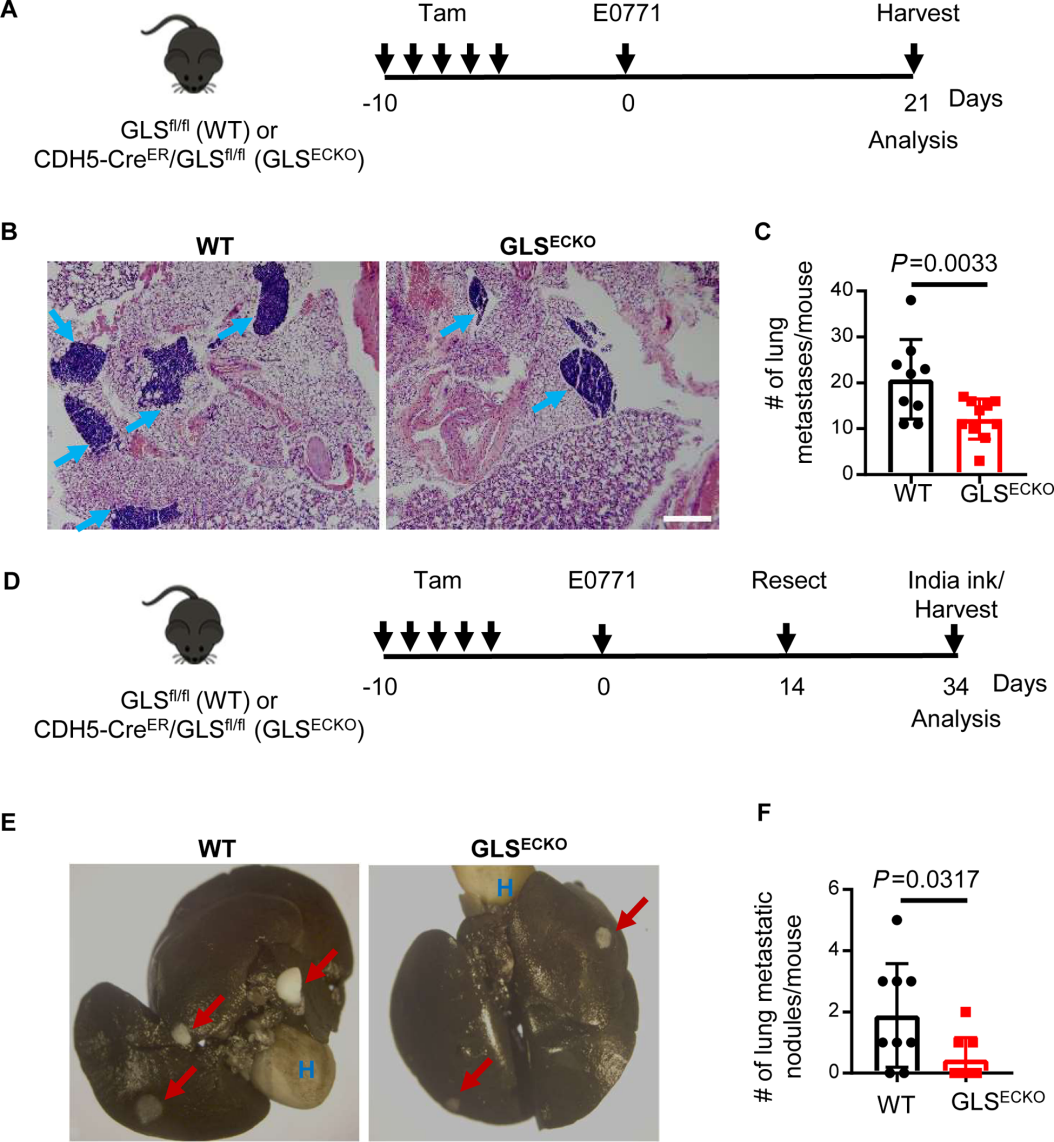
Loss of vascular endothelial GLS reduces breast cancer metastasis. **A,** A schematic diagram showing the experimental procedure of tamoxifen treatment (5×: five times) and E0771 mammary fat pad implantation. **B,** Representative histologic images of H&E-stained lung sections from WT and GLS^ECKO^ mice (*n* = 9–10). Scale bar: 200 μm. Blue arrows denote lesions. **C,** Quantification of metastatic lung lesions per mouse in WT versus GLS^ECKO^ (*n* = 9–10 mice per group). **D,** A schematic diagram showing the experimental procedure of tamoxifen treatment (five times), E0771 mammary fat pad implantation, resection, and India ink Injection. **E,** Representative images of India ink–infused lungs from WT versus GLS^ECKO^ mice following primary tumor resection. Red arrows indicate metastatic foci. H (blue) indicates the position of heart. **F,** Quantification of metastatic nodules in lungs of WT versus GLS^ECKO^ (*n* = 9 mice per group). Data in **C** and **F** are presented as mean ± SEM from two or three independent experiments. *P* values were determined by two-tailed unpaired Student *t* test.

We assessed lung metastasis at the end of studies, 3 weeks after E0771 tumor implantation. There were no surface metastatic lesions observed; however, serial section (*n* = 5/lung) of lungs revealed that the numbers of microscopic metastases in WT mice was significantly greater than that detected in GLS^ECKO^ mice (Fig. 2A–C). In addition, we examined the area of each metastatic lesion but did not observe any significant difference between WT or GLS^ECKO^ mice, suggesting that loss of GLS in the host vasculature may reduce dissemination of cells to the lung rather than impact outgrowth of tumor cells once they arrive at the lungs. To be rigorous, we repeated these studies with a modified approach, removing primary tumors at day 14 and assessing lung surface metastatic lesions 20 days following tumor resection (Fig. 2D). WT mice displayed significantly more visible metastatic lung nodules compare with those in GLS^ECKO^ animals (Fig. 2E and F). Collectively, these results showed that loss of GLS in the host endothelium inhibited primary tumor growth and lung metastasis.

### Endothelial GLS Deletion Reduces Tumor Vascular Density and Normalizes Tumor Vessels

**FIGURE 3:**
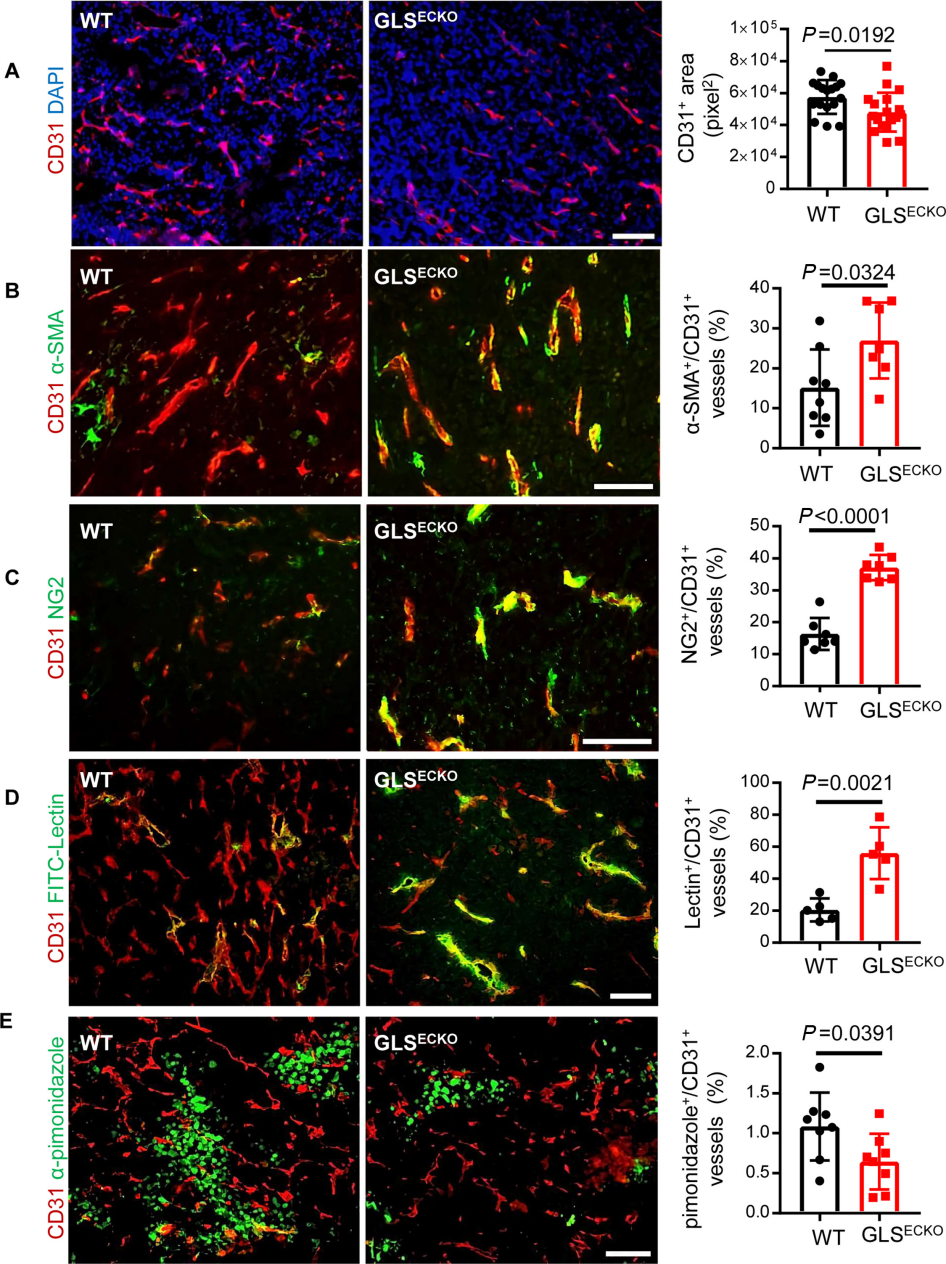
Endothelial GLS deletion reduces tumor vascular density and normalizes tumor vessels. **A,** Representative immunofluorescent images and quantification of CD31^+^ vessels (red), DAPI (nuclei, blue) in E0771 tumors harvested from WT control and GLS^ECKO^ mice (*n* = 10–12 mice per group). Scale bar: 100 μm. **B,** Representative immunofluorescent images and quantification of pericyte coverage (α-SMA; green) on tumor vessels (red). Pericyte coverage is presented as percentage of α-SMA^+^/CD31^+^ blood vessels (yellow), (*n* = 7–8 mice per group). Scale bar: 100 μm. **C,** Representative immunofluorescent images and quantification of pericyte coverage on tumor vessels using NG2 marker (green). *n* = 7 per group. Scale bar: 50 μm. **D,** Representative immunofluorescent images and quantification of tumor vessel perfusion. Functional blood vessels were assessed by perfusion of FITC-lectin (green) in CD31^+^ tumor blood vessels (red). Vessel perfusion is presented as percentage of Lectin^+^ area within the CD31^+^ vessels (*n* = 5 per group). Scale bar: 100 μm. **E,** Representative immunofluorescent images and quantification of pimonidazole^+^ hypoxic regions within E0771 tumors. Hypoxia area was assessed by injecting hydroxyprobe into tumor-bearing mice. Hypoxic regions (green) and CD31^+^ blood vessels (red) are shown within the tumor (*n* = 8 mice per group). Scale bar: 100 μm. All data are presented as mean ± SEM from two or three independent experiments. *P* values were determined by two-tailed unpaired Student *t* test.

To determine the impact of endothelial GLS deletion specifically on vasculature, we first assessed vascular density and maturation in multiple organs in adult animals without tumors. We did not observe significant differences between WT and GLS^ECKO^ mice in the vasculature of mammary gland, lung, kidney, and liver (Supplementary Fig. S2A–S2D). To investigate whether GLS loss affects tumor vasculature, we analyzed tumor sections from tumors harvested 2 to 3 weeks after implantation. Compared with those from WT animals, tumors derived from GLS^ECKO^ mice had reduced microvascular density as measured by CD31 staining, suggesting that loss of GLS in the endothelium reduces angiogenic sprouts in tumors (Fig. 3A). To assess tumor vessel integrity, CD31-positive vessels were costained with pericyte markers, α-SMA or NG2. We observed that α-SMA^+^ or NG2^+^ pericyte coverage on tumor vessels was significantly increased in GLS^ECKO^ tumors (Fig. 3B and C), indicative of tumor vessel maturation and structural integrity.

We next evaluated tumor vessel functionality by tracing intravenously injected fluorescently labeled lectin (FITC-Lectin) as a measure of vessel perfusion. Lectin-perfused vessel areas were markedly elevated in tumors of GLS^ECKO^ hosts compared with those of WT host (Fig. 3D). Furthermore, we examined hypoxic areas in the tumor tissue by intravenously injecting pimonidazole hydrochloride, a bioreductive chemical probe which becomes activated at low oxygen conditions (23), into E0771 tumor-bearing WT and GLS^ECKO^ mice. We observed a decrease in hypoxic tumor areas in GLS^ECKO^ E0771 tumors compared with WT tumors (Fig. 3E), suggesting that GLS^ECKO^ tumors were less hypoxic than the WT. Taken together, these data suggest that endothelial GLS loss leads to tumor vessel normalization.

### Decreased Leptin in GLS^ECKO^ Tumors and Leptin Treatment Rescued Tumor Growth Defects in GLS^ECKO^ Mice

**FIGURE 4:**
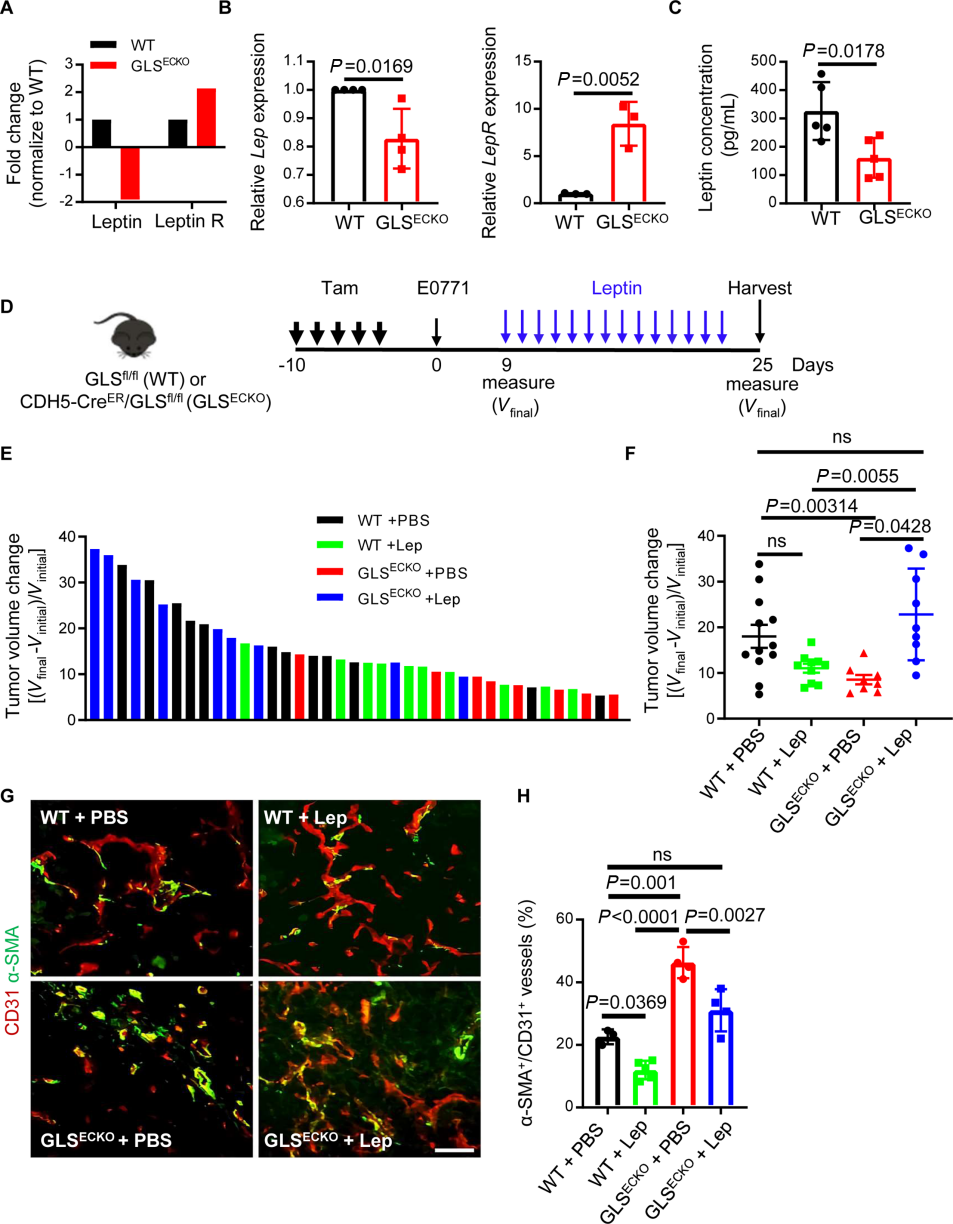
Leptin treatment rescues tumor growth defects in GLS^ECKO^ mice. **A,** Cytokine array showing differentially expressed leptin and leptin receptor in WT versus GLS^ECKO^ E0771 tumor lysates. **B,** Leptin and Leptin receptor mRNA expression was measured by qRT-PCR (*n* = 3–4) in E0771 tumor cells isolated from WT versus GLS^ECKO^ mice. **C,** Leptin ELISA from whole tumor lysates of WT versus GLS^ECKO^ (*n* = 5 mice per group). **D,** A schematic diagram of experimental design with E0771 tumor allograft and leptin treatment (1 mg/kg). **E,** Tumor volume change of WT and GLS^ECKO^ mice treated with leptin or PBS control (*n* = 8–13) calculated as [(*V*_final_ − *V*_initial_)/*V*_initial_]. *V*_final_ = volume on last day of treatment; *V*_initial_ = volume on first day of treatment. **F,** Quantification of tumor volume change. Representative images (**G**) and quantification of α-SMA^+^CD31^+^ vessels (**H**), showing tumor vessel normalization reversal following leptin treatment. (Scale bar: 50 μm). All data are presented as ± SEM. *P* values were determined by two-tailed unpaired Student *t* test (**B** and **C**), one-way ANOVA with Tukey multiple comparisons test (**F** and **H**).

Tumor vessel normalization was previously shown to enhance the number and effector functions of infiltrating lymphocytes (24–26). However, we detected neither significant changes in tumor-infiltrating CD8^+^ cytotoxic T cells or CD4^+^ Th cells nor differences in natural killer cells, macrophages, or myeloid-derived suppressor cells between WT and GLS^ECKO^ in both E0771 and MMTV-PyMT tumors (Supplementary Fig. S3A–S3L). As a first step to determine the molecular mechanism underlying the effect of GLS deficiency on tumor growth phenotypes, E0771 tumor lysates harvested from WT or GLS^ECKO^ mice were assessed for changes in cytokine and chemokine production. While several cytokines appear to increase to varying degrees, we found that leptin was the only factor that is decreased in GLS^ECKO^ tumors (Fig. 4A; Supplementary Fig. S4A). Notably, this decrease in leptin (Lep) was accompanied by an increase in leptin receptor, suggesting that leptin signaling may be altered in GLS^ECKO^ tumors (Fig. 4A). The decrease of leptin and increase of leptin receptor in GLS^ECKO^ tumors was further confirmed by quantitative RT-PCR analyses on isolated tumor cells (Fig. 4B). Further validation of Lep in whole tumor lysate by ELISA with additional independent tumor samples showed that the levels of leptin were significantly decreased in tumors derived from GLS^ECKO^ mice relative to WT mice (Fig. 4C). Consistent with cytokine array data and ELISA results, we also detected a reduction in leptin staining in the GLS^ECKO^ tumors compared with WT tumors by IHC on tumor sections (Supplementary Fig. S4B). Together, these results suggest that loss of GLS in tumor endothelium affects leptin levels in tumors.

Leptin is a protein hormone that primarily functions to regulate appetite and energy expenditure (27–30). The leptin-leptin receptor signaling is also known to play a critical role in cancer progression, including cell proliferation, metastasis, angiogenesis, and chemoresistance (31–34). We reasoned that increased leptin receptor expression in tumors from GLS^ECKO^ mice may render these tumors sensitive to leptin treatment. To test this hypothesis and to determine whether tumor growth is dependent on leptin, E0771 tumors were implanted into tamoxifen-treated mice, and the tumor-bearing animals were administered with recombinant leptin or vehicle controls (Fig. 4D). Addition of leptin rescued defective tumor growth in GLS^ECKO^ hosts, compared with those treated with vehicle controls (Fig. 4E and F). In addition, leptin treatment reverses the vascular normalization phenotype in GLS^ECKO^ tumors (Fig. 4G and H). Collectively, these data suggest that endothelial GLS deletion reduces tumor growth, at least in part, through reduced leptin levels, consistent with previously described important roles of leptin in breast cancer (31, 35).

### Loss of Endothelial GLS Promotes Delivery of Chemotherapeutic Agents

**FIGURE 5:**
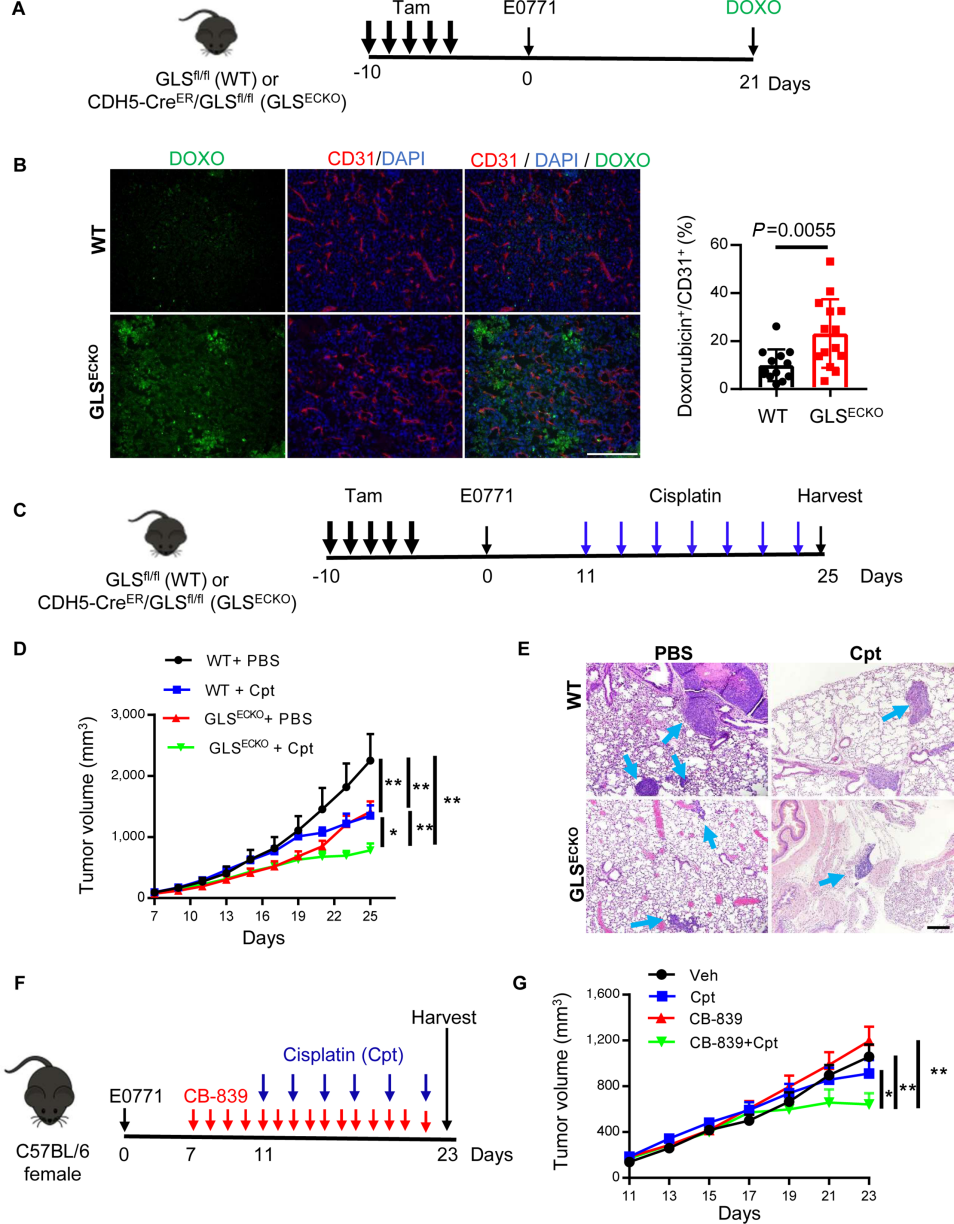
Loss of endothelial GLS enhances the efficacy of chemotherapeutic agents. **A,** A schematic diagram showing the experimental procedure of tamoxifen (Tam) treatment (five times), tumor implantation, and DOXO treatment. **B,** Representative images and quantification of DOXO autofluorescence in E0771 tumors grown in WT versus GLS^ECKO^ mice (*n* = 13–14 mice per group). The doxorubicin^+^ area (DOXO green) is presented as a percentage of total nuclei, DAPI^+^ (blue). Scale bar: 200 μm. **C,** A schematic diagram showing the experimental procedure of tamoxifen treatment (five times), tumor inoculation, and Cpt treatment. **D,** Growth curve of E0771 tumors grown in WT versus GLS^ECKO^ mice treated with either vehicle control (PBS) or Cpt, *n* = 6–7 mice per group. **, *P* ≤ 0.01. *, *P* ≤ 0.05. **E,** Representative histologic images of H&E-stained lung sections of Cpt and control treated WT and GLS^ECKO^ tumors (*n* = 6–7 mice per group). Blue arrows indicate metastatic foci. Scale bar: 200 μm. Quantification of metastatic lung lesions per mouse in WT versus GLS^ECKO^ treatment groups is shown in Supplementary Fig. S5. **F,** A schematic diagram showing the experimental procedure of tamoxifen treatment, tumor inoculation, CB-839, and Cpt treatment. **G,** Growth curve of E0771 tumors upon treatment with vehicle (Veh), CB-839 (50 mg/kg by i.p, daily), and Cpt (4 mg/kg by i.p, every other day), alone or in combination with CB839 (*n* = 6–7 per group). All data are presented as mean ± SEM from two or three independent experiments. *P* values were determined by two-tailed unpaired Student *t* test (**B**), two-way ANOVA with Tukey multiple comparison test (**D** and **G**). **, *P* ≤ 0.01. *, *P* ≤ 0.05.

On the basis of the significant increase in tumor vessel perfusion observed in GLS^ECKO^ hosts, we assessed the impact of endothelial GLS loss on chemotherapeutic drug delivery. DOXO was initially used because its autofluorescence property enables direct monitoring of the drug's penetration into the tumor. DOXO was intravenously injected 3 weeks following inoculation of E0771 tumors into WT and GLS^ECKO^ mice (Fig. 5A). We observed an increase in DOXO accumulation in GLS^ECKO^ tumor sections compared with their WT control counterparts, suggesting improved delivery of the chemotherapeutic drug in GLS^ECKO^ tumors (Fig. 5B). Because DOXO has been shown to be less potent in triple-negative breast cancer PDX and E0771 models than platinum-based therapies (21, 36, 37), we examined the antitumor effect of Cpt chemotherapy in GLS^ECKO^ tumor model (Fig. 5C). As expected (21), tumor growth was significantly reduced by loss of GLS in tumor endothelium or Cpt treatment of WT animals (Fig. 5D). However, a combination of GLS deletion in host vessels with Cpt administration further decreased tumor growth relative to WT tumor growth (∼4-fold at day 25; Fig. 5D). H&E sections from the lungs of these mice also showed reduced metastatic lung lesions in GLS^ECKO^ mice treated with Cpt compared with GLS^ECKO^ animals (Fig. 5E; Supplementary Fig. S5). Taken together, these findings demonstrate that loss of GLS in the endothelium enhances the delivery and efficacy of chemotherapy.

### Pharmacologic Inhibition of GLS Enhance Efficacy of Chemotherapeutic Agents

To investigate clinical relevance of GLS inhibition in the tumor blood vessels, we tested whether pharmacologic inhibition of GLS can improve efficacy of chemotherapy. E0771 tumor cells were implanted into WT C57BL/6 female mice. When tumors grew to approximately 60 mm^3^ (day 7), mice were randomized and treated with vehicle or CB-839 (50 mg/kg) by intraperitoneal injection every day for 16 days (Fig. 5F). On day 11, animals were further randomized to receive either PBS or Cpt (4 mg/kg) every other day for a total of six doses (Fig. 5F). CB-839 monotherapy did not significantly reduce tumor growth but a combination of CB-839 and Cpt significantly inhibited tumor growth greater than either CB-839 or Cpt treatment alone (Fig. 5G), suggesting that pharmacologic inhibition of GLS enhances efficacy of chemotherapy, which may be leveraged to improve clinic outcome in glutamine-addicted tumors.

## Discussion

Rapidly growing tumors require blood vessels to supply oxygen and nutrients to meet their bioenergetic and biosynthetic requirements. Cancer cells secrete angiogenic factors that promote the proliferation of tumor vascular ECs, resulting in a tortuous, leaky, and chaotic networks of endothelial tubes (38, 39). This angiogenic process is an energy and biomass-demanding process (40). Indeed, proliferating angiogenic sprouts in tumors favor glycolysis over OXPHOS, upregulating GLUT-1 and LDH-A, and PFKFB3, all of which are key glycolytic regulators in ECs (5, 41). Inhibition of PFKFB3 either genetically or pharmacologically normalized tumor blood vessels, leading to suppression of tumor metastasis and improvement of drug delivery (6). Glutamine is also required for normal EC proliferation, migration, and sprouting (7, 8), but its role in tumor vasculature remains unclear. Given that GLS2 expression levels are low in ECs (ref. 16; Supplementary Fig. S6A–S6H), we focused on the effects of GLS1 (GLS). We showed that loss of GLS in tumor endothelium inhibited vascular density, improved perivascular cell coverage, enhanced tissue perfusion, and reduced tumor hypoxia, leading to reduced tumor growth and metastasis and improvement of drug delivery. These data highlight the important role of glutamine in tumor blood vessel and inhibition of glutamine metabolism in the tumor vasculature may sensitize tumors to various therapeutic agents.

We demonstrated that GLS loss in tumor endothelium leads to decreased leptin but increased leptin receptor levels in E0771 tumors. In addition to its traditional role in regulating appetite and energy expenditure, leptin signaling is implicated in promoting tumor cell proliferation and migration (42–44). Although adipose cells are known to be major source of leptin (45, 46), it is currently unclear how glutamine metabolism in EC precisely regulates leptin production within the mammary tumor microenvironment. In addition to leptin produced by tumor cells (Fig. 4B), we speculate that interaction between ECs and adipose cells in the tumor microenvironment accounts for dysregulation of leptin. This is based on our observation that the defective tumor growth phenotype was only observed when tumor cells were injected into the mammary fat pad, but not when the tumor cells were implanted subcutaneously with LLC and B16F10 cell lines (Supplementary Fig. S7A and S7B). The reciprocal increase in leptin receptor in GLS^ECKO^ tumors may represent a compensatory response to decreased leptin level, possibly enhancing the sensitivity of tumors to leptin treatment. Indeed, administration of exogenous recombinant leptin rescued tumor growth defects and reverses the vascular normalization phenotype in GLS^ECKO^ mice, suggesting that leptin is one mechanism through which endothelial glutamine metabolism controls tumor growth.

How loss of GLS in ECs could affect leptin levels in the tumors is currently unclear. We did not see changes in VEGF/VEGFR2 expression between WT or GLS^ECKO^ vascular ECs (Supplementary Fig. S6A–S6H). However, it was previously shown that hypoxia through hypoxia-inducible factor-1α induced leptin mRNA and protein expression in breast cancer cells (47). Thus, reduced leptin level is consistent with lower hypoxia induced by increased blood vessel normalization in GLS^ECKO^ tumor. These discoveries open exciting opportunities for future investigations on how glutamine metabolism in the endothelium affects leptin and leptin receptor in breast cancer.

The tumor microenvironment is a diverse landscape, containing tumor cells, immune cells, ECs, and fibroblasts, among others (48). Normalization of tumor blood vessels has been recognized recently to recruit tumor-infiltrating lymphocytes and enhance antitumor immune responses (26, 49–51). Although loss of GLS in tumor endothelium normalized tumor vessels, we did not observe consistent changes in lymphocyte numbers or effector function within the time frame in our model systems (Supplementary Fig. S3). Previous studies demonstrated a positive feedback loop between CD4^+^ Th1 cells and vessel normalization, involving cell–cell interaction, cytokine production and pericyte maturation, and coverage (24). Our data do not exclude the possibility of a role of endothelial GLS in regulating recruitment and function of tumor-infiltrating lymphocytes. However, it is conceivable that in addition to tumor vessel normalization, other cytokines or factors in the microenvironment may be required for immunostimulatory reprogramming in cancer.

Addiction to glutamine in many tumors led to targeting glutamine metabolism as a mean to therapeutically treat these aggressive tumors. For example, the GLS inhibitor CB-839 is undergoing clinical trials for numerous solid tumors. Although as a single-agent CB-839 has limited efficacy, our results indicate that targeting endothelial GLS genetically leads to tumor vessel normalization and increased drug delivery. Thus, CB-839 may be a good candidate for combination therapy with chemotherapeutic agents or immune checkpoint inhibitors. Indeed, our data show that CB-839 improve efficacy of Cpt antitumor effect. Given the complex interplay between oncogenic signaling and metabolic rewiring among different cell types in the tumor microenvironment, elucidating how glutamine metabolism in ECs impacts tumor growth is of great importance and could provide opportunities for therapeutic intervention.

## Supplementary Material

Supplementary RNA-seq Data RNA1 Excel file showing processed transcriptomics data from RNA-seq.

Supplementary Fig. S1 This figure shows immunofluorescence images of increased apoptosis (Cleaved- Caspase 3) in GLSECKO tumors compared to WT while there was no significant change in proliferation (Ki-67). Quantification of these data were shown in Figure 1G and 1H.

Supplementary Fig. S2 This figure shows immunofluorescence images and quantification of CD31 and α-SMA in mammary gland, kidney, Lung and Liver tissues from non-tumor bearing mice after they were treated with tamoxifen.

Supplementary Fig. S3 This figure shows flow cytometry data from GLSECKO compared to WT from E0771 and MMTV-PyMT tumor models

Supplementary Fig. S4 This figure shows Cytokines array data of whole E0771 tumor lysate and immunohistochemistry of leptin staining on GLSECKO versus WT tumor sections.

Supplementary Fig. S5 This figure shows the quantification of lung metastases in WT or GLSECKO mice treated with Cisplatin. Representative images were shown in Figure 5E.

Supplementary Fig. S6 This figure shows volcano plots of differentially expressed genes in GLSECKO versus WT endothelial cell, differentially enriched pathways up and down, Gene set enrichment analysis, -log FPKM of Gls, Gls2, angiogenic genes, qRT-PCR of leptin and angiogenic genes in GLSECKO versus WT.

Supplementary Fig. S7 This figure shows tumor growth curves of B16F10 and LLC-GFP-Luc tumors grown subcutaneously in GLSECKO versus WT mice

Supplementary Materials and Methods1 This is additional Supplementary Materials and Methods including tables.

Supplementary qPCR Data qPCR1 Excel file showing qPCR Data.
