# Supplementary material for: Loss of Vascular Endothelial Glutaminase Inhibits Tumor Growth and Metastasis, and Increases Sensitivity to Chemotherapy
Source: Cancer Res Commun. 2022 Jul 21;2(7):694–705. doi: 10.1158/2767-9764.CRC-22-0048 (PMC9645801; doi:10.1158/2767-9764.CRC-22-0048)
Supplement: Supplementary Fig. S1 — This figure shows immunofluorescence images of increased apoptosis (Cleaved- Caspase 3) in GLSECKO tumors compared to WT while there was no significant change in proliferation (Ki-67). Quantification of these data were shown in Figure 1G and 1H. [file crc-22-0048-s02.pdf]

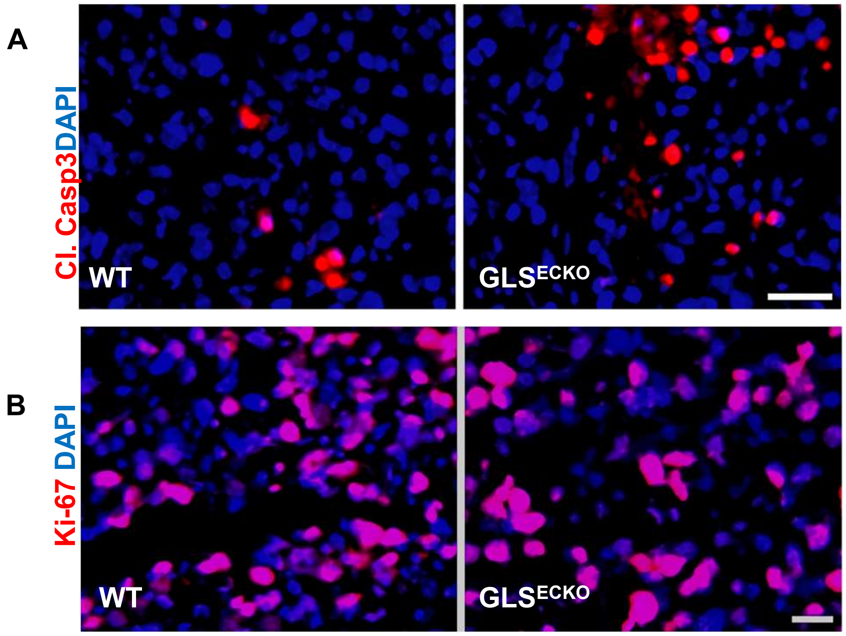

Supplementary Fig. S1: **Loss of GLS in the endothelium increases apoptosis but not cell proliferation.** (**A**) Representative immunofluorescence images of cleaved-caspase 3 (red) and DAPI (blue) in WT versus GLS^{ECKO} tumors. Scale bar: 50 µm. (**B**) Representative immunofluorescence images of Ki-67 (red) and DAPI (blue) in WT versus GLS^{ECKO} tumors. Scale bar: 20 µm.
