# Supplementary material for: Loss of Vascular Endothelial Glutaminase Inhibits Tumor Growth and Metastasis, and Increases Sensitivity to Chemotherapy
Source: Cancer Res Commun. 2022 Jul 21;2(7):694–705. doi: 10.1158/2767-9764.CRC-22-0048 (PMC9645801; doi:10.1158/2767-9764.CRC-22-0048)
Supplement: Supplementary Fig. S2 — This figure shows immunofluorescence images and quantification of CD31 and α-SMA in mammary gland, kidney, Lung and Liver tissues from non-tumor bearing mice after they were treated with tamoxifen. [file crc-22-0048-s03.pdf]

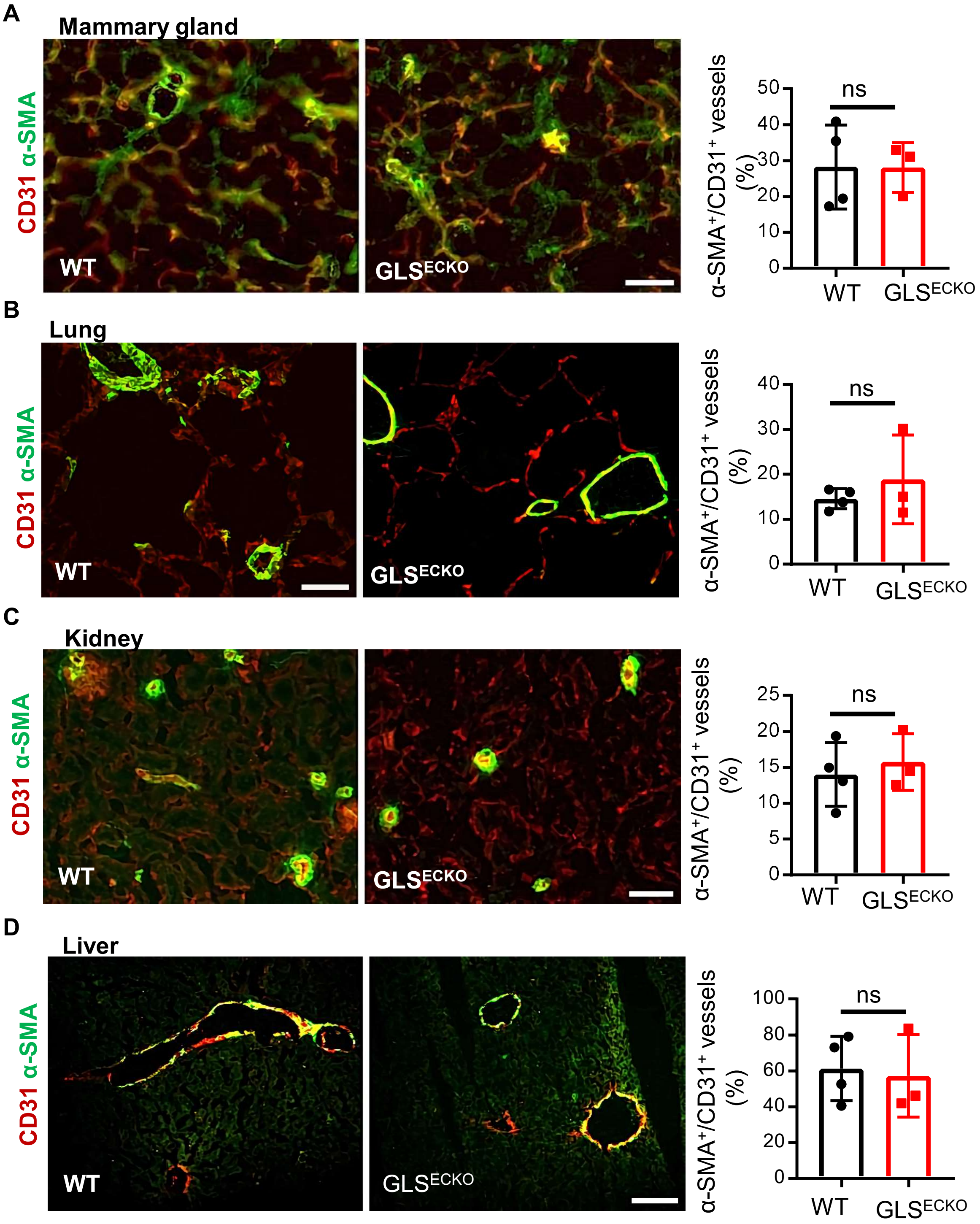

Supplementary Fig. S2: Loss of GLS in tumor-free endothelium does not affect the vasculature of adult animals. WT and GLS^{ECKO} mice were treated with tamoxifen for 5 consecutive days to induce EC-specific loss of GLS. One week following the last tamoxifen treatment, the mammary gland (**A**), Lungs (**B**), Kidneys (**C**) and Liver (**D**) were harvested and co-stained with CD31 (red) and α -SMA (green) to assess vascular integrity. n=3-4 mice per group. Scale bar: 50 µm (**A**, **B**, **C**) and 100 µm (**D**).
