# Supplementary material for: Loss of Vascular Endothelial Glutaminase Inhibits Tumor Growth and Metastasis, and Increases Sensitivity to Chemotherapy
Source: Cancer Res Commun. 2022 Jul 21;2(7):694–705. doi: 10.1158/2767-9764.CRC-22-0048 (PMC9645801; doi:10.1158/2767-9764.CRC-22-0048)
Supplement: Supplementary Fig. S3 — This figure shows flow cytometry data from GLSECKO compared to WT from E0771 and MMTV-PyMT tumor models [file crc-22-0048-s04.pdf]

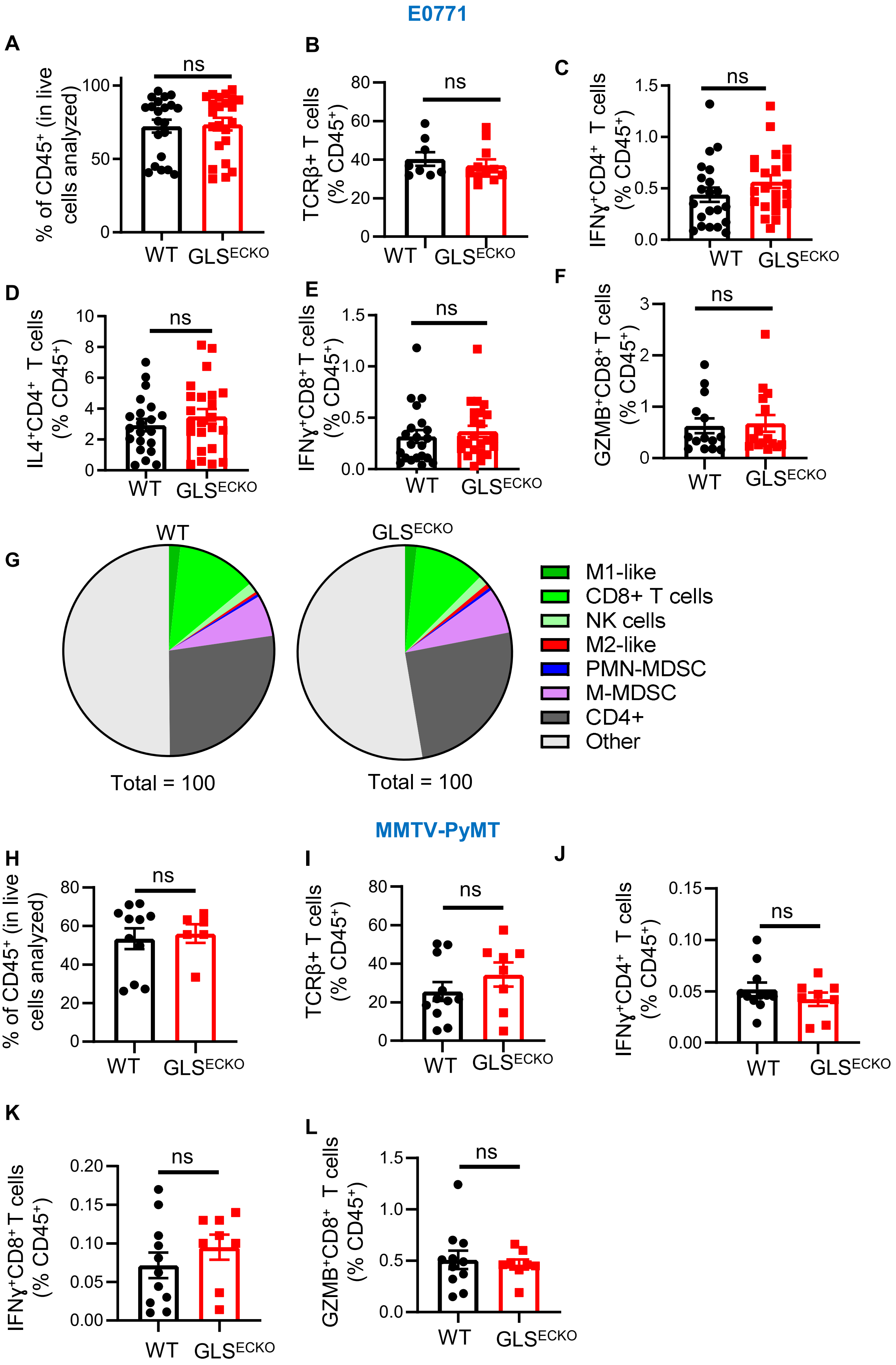
E0771
A
% of CD45+ (in live cells analyzed)
ns
WT
GLSECKO
B
TCRβ+ T cells (% CD45+)
C
IFNγ+CD4+ T cells (% CD45+)
D
IL4+CD4+ T cells (% CD45+)
E
IFNγ+CD8+ T cells (% CD45+)
F
GZMB+CD8+ T cells (% CD45+)
G
M1-like
CD8+ T cells
NK cells
M2-like
PMN-MDSC
M-MDSC
CD4+
Other
Total = 100
MMTV-PyMT
H
I
J
K
L
GZMB+CD8+ T cells (% CD45+)

Supplementary Fig. S3: Endothelial GLS deletion does not appear to affect tumor infiltrating immune cells. E0771 tumors (**A-G**) or PyMT tumors (**H-L**) were harvested from WT or GLS^{ECKO} mice. Tumor infiltrating immune cells were quantified by flow cytometric analyses, including $CD45^{+}$ cells (**A** and **H**), T cells (**B** and **I**), Th1 and Th2 CD4 T cells (**C, D,** and **J**), $IFN\gamma^{+}$ and $GZMB^{+}$ CD8 T cells (**E, F, K**, and **L**). Percentages of different cell types within the E0771 tumors are presented in the pie chart (G). All data are presented as mean ± SEM from 2 of 3 independent experiments. ns, not significant as determined by two-tailed unpaired Student's t-test.
