# Supplementary material for: Loss of Vascular Endothelial Glutaminase Inhibits Tumor Growth and Metastasis, and Increases Sensitivity to Chemotherapy
Source: Cancer Res Commun. 2022 Jul 21;2(7):694–705. doi: 10.1158/2767-9764.CRC-22-0048 (PMC9645801; doi:10.1158/2767-9764.CRC-22-0048)
Supplement: Supplementary Fig. S4 — This figure shows Cytokines array data of whole E0771 tumor lysate and immunohistochemistry of leptin staining on GLSECKO versus WT tumor sections. [file crc-22-0048-s05.pdf]

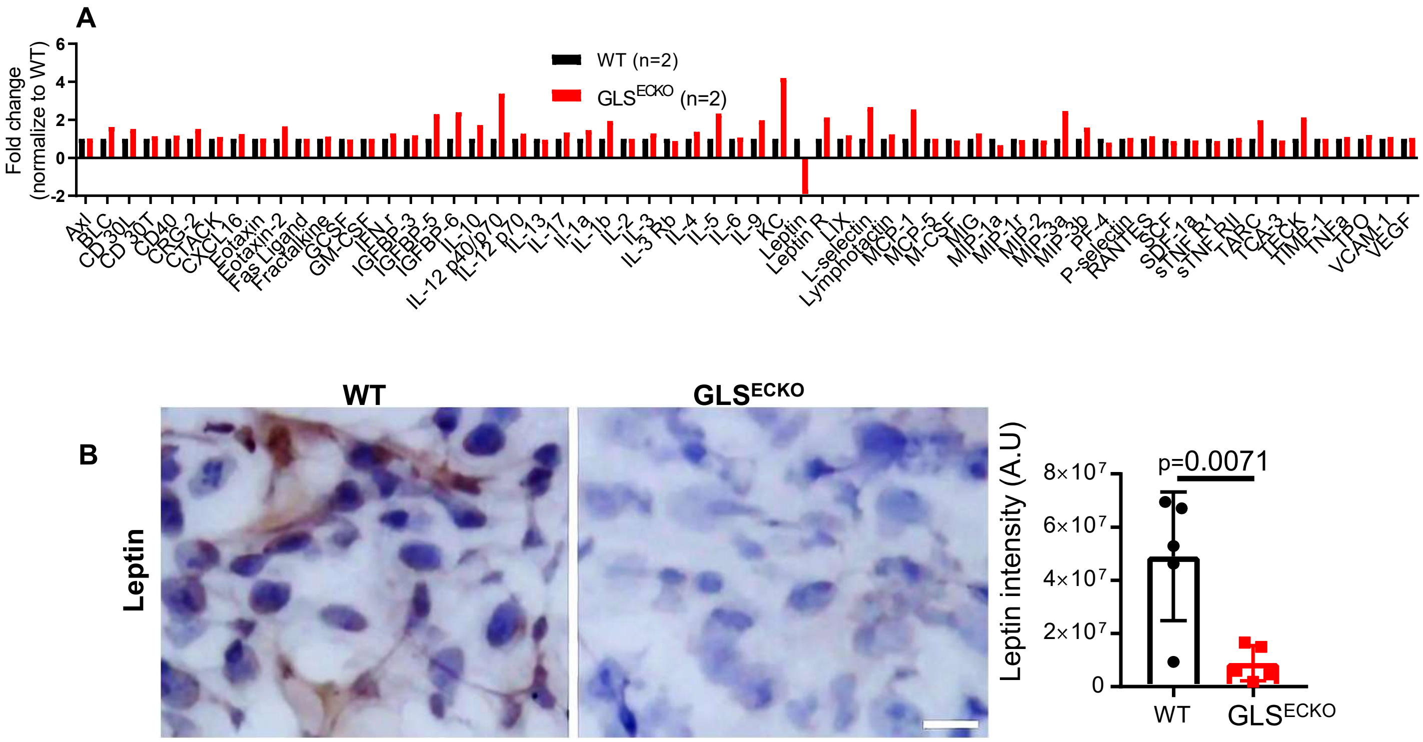

Supplementary Fig. S4: (**A**) Cytokine array showing differentially expressed proteins in WT versus GLS^{ECKO} tumor lysates, including Leptin and Leptin receptor. (**B**) Representative immunohistochemistry images and quantification showing decrease leptin expression in tumor sections of GLS^{ECKO} compared to WT. n=5 mice per group. Scale bar: 50 μm
