# Supplementary material for: Loss of Vascular Endothelial Glutaminase Inhibits Tumor Growth and Metastasis, and Increases Sensitivity to Chemotherapy
Source: Cancer Res Commun. 2022 Jul 21;2(7):694–705. doi: 10.1158/2767-9764.CRC-22-0048 (PMC9645801; doi:10.1158/2767-9764.CRC-22-0048)
Supplement: Supplementary Fig. S5 — This figure shows the quantification of lung metastases in WT or GLSECKO mice treated with Cisplatin. Representative images were shown in Figure 5E. [file crc-22-0048-s06.pdf]

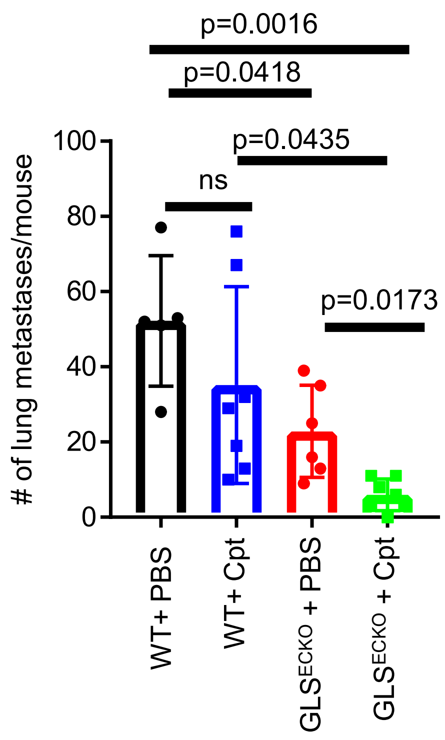

Supplementary Fig. S5 : Quantification of lung metastases in WT or GLS^{ECKO} mice treated with Cisplatin Tumor implantation and cisplatin treatment are as Figure 5. At the end of studies, lungs were harvested and metastases in lung were quantified in 5 sections/mouse (n=6-7 mice/group). $^{**}P \leq 0.01$. $^{*}P \leq 0.05$. one-way ANOVA with Dunnett's correction. ns: not significant.
