# Supplementary material for: Loss of Vascular Endothelial Glutaminase Inhibits Tumor Growth and Metastasis, and Increases Sensitivity to Chemotherapy
Source: Cancer Res Commun. 2022 Jul 21;2(7):694–705. doi: 10.1158/2767-9764.CRC-22-0048 (PMC9645801; doi:10.1158/2767-9764.CRC-22-0048)
Supplement: Supplementary Fig. S6 — This figure shows volcano plots of differentially expressed genes in GLSECKO versus WT endothelial cell, differentially enriched pathways up and down, Gene set enrichment analysis, -log FPKM of Gls, Gls2, angiogenic genes, qRT-PCR of leptin and angiogenic genes in GLSECKO versus WT. [file crc-22-0048-s07.pdf]

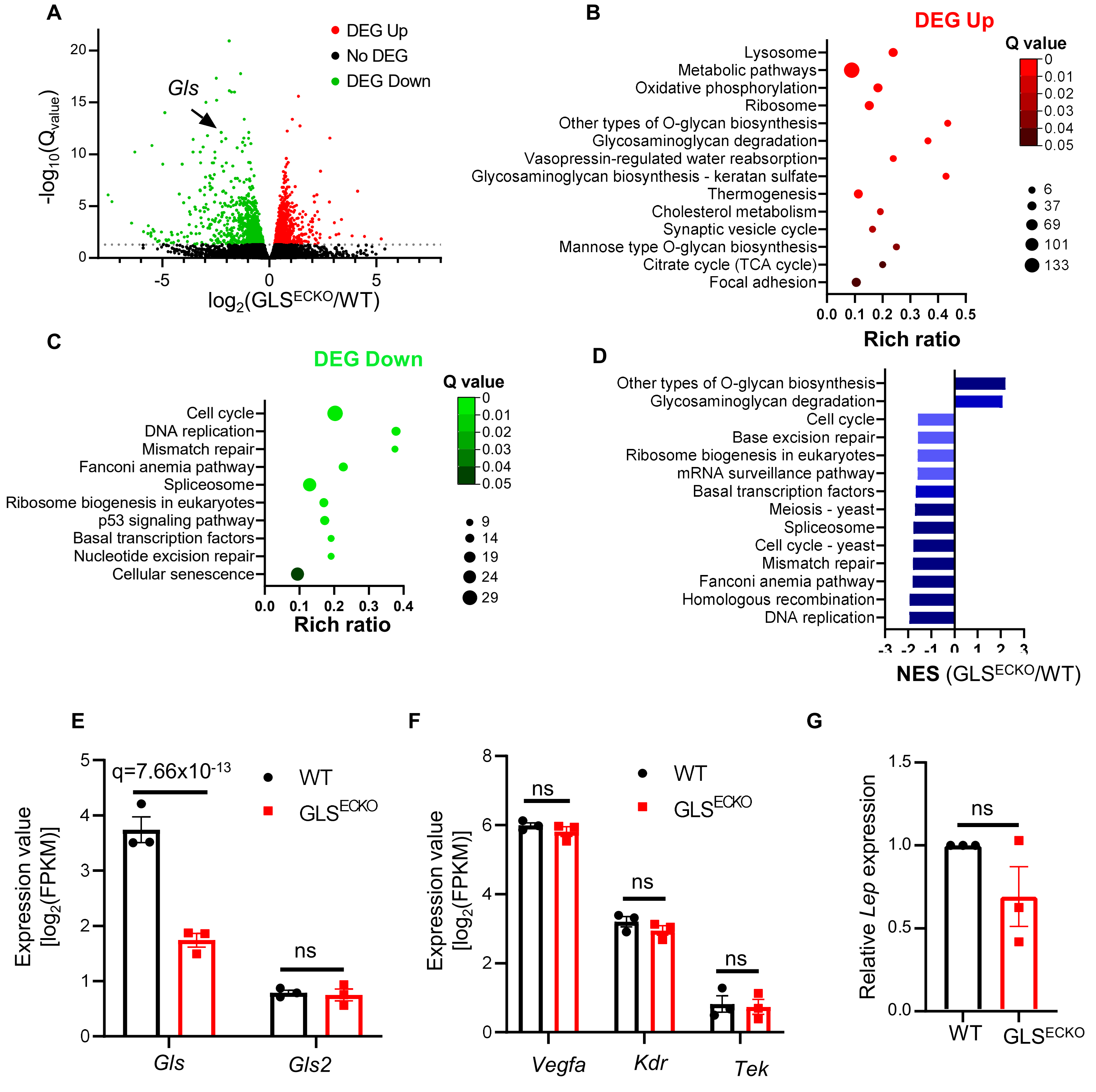
A
DEG Up
No DEG
DEG Down
Gls
$-\log_{10}(Q_{value})$
$\log_2(GLS^{ECKO}/WT)$
B
DEG Up
Q value
Lysosome
Metabolic pathways
Oxidative phosphorylation
Ribosome
Other types of O-glycan biosynthesis
Glycosaminoglycan degradation
Vasopressin-regulated water reabsorption
Glycosaminoglycan biosynthesis - keratan sulfate
Thermogenesis
Cholesterol metabolism
Synaptic vesicle cycle
Mannose type O-glycan biosynthesis
Citrate cycle (TCA cycle)
Focal adhesion
Rich ratio
C
DEG Down
Q value
Cell cycle
DNA replication
Mismatch repair
Fanconi anemia pathway
Spliceosome
Ribosome biogenesis in eukaryotes
p53 signaling pathway
Basal transcription factors
Nucleotide excision repair
Cellular senescence
Rich ratio
D
Other types of O-glycan biosynthesis
Glycosaminoglycan degradation
Cell cycle
Base excision repair
Ribosome biogenesis in eukaryotes
mRNA surveillance pathway
Basal transcription factors
Meiosis - yeast
Spliceosome
Cell cycle - yeast
Mismatch repair
Fanconi anemia pathway
Homologous recombination
DNA replication
NES (GLS^{ECKO}/WT)
E
$q=7.66x10^{-13}$
WT
GLS^{ECKO}
ns
Expression value
$[\log_2(FPKM)]$
Gls
Gls2
F
ns
WT
GLS^{ECKO}
Expression value
$[\log_2(FPKM)]$
Vegfa
Kdr
Tek
G
ns
Relative Lep expression
WT
GLS^{ECKO}

Supplementary Fig. S6: RNA seq analysis of WT and GLS^{ECKO} endothelial cells. Murine pulmonary microvascular endothelial cells (MPMECs) were isolated from GLS f/f mice and transduced with either Ad-Cre or Ad-control. Deletion of GLS in MPMECs was confirmed by western blot analysis. RNAs were isolated from either WT (Ad-control) or GLS^{ECKO} (Ad-Cre) endothelial cells and sent to BGI Inc. for sequencing and analysis using the Dr. Tom software. (**A**) Volcano plot of differentially expressed genes (DEG) in GLS^{ECKO} versus WT. Red and green dots mark the upregulated and downregulated genes respectively in GLS^{ECKO} compared to WT (n= 3 per group, FDR q-value < 0.05). (**B-C**) Bubble chart showing KEGG enrichment analysis of DEGs up (**B**) and DEGs down (**C**) comparing GLS^{ECKO} with WT. Circle size represents the gene number while circle color represents the value of $-\log_{10}(q_{value})$. (**D**) The mean normalized enrichment score (NES) of the gene set enrichment analysis (GSEA) comparing to GLS^{ECKO} to WT are represented by each bar. NES <0 and NES >0 represent downregulation and upregulation of specified pathways in GLS^{ECKO} versus WT respectively. (**E-G**) Expression values of Gls2, Vegfa, Kdr (Vegfr2), Tek (Tie2), and Leptin (Lep) in GLS^{ECKO} compared to WT. ns: not significant.
