# Supplementary material for: Loss of Vascular Endothelial Glutaminase Inhibits Tumor Growth and Metastasis, and Increases Sensitivity to Chemotherapy
Source: Cancer Res Commun. 2022 Jul 21;2(7):694–705. doi: 10.1158/2767-9764.CRC-22-0048 (PMC9645801; doi:10.1158/2767-9764.CRC-22-0048)
Supplement: Supplementary Fig. S7 — This figure shows tumor growth curves of B16F10 and LLC-GFP-Luc tumors grown subcutaneously in GLSECKO versus WT mice [file crc-22-0048-s08.pdf]

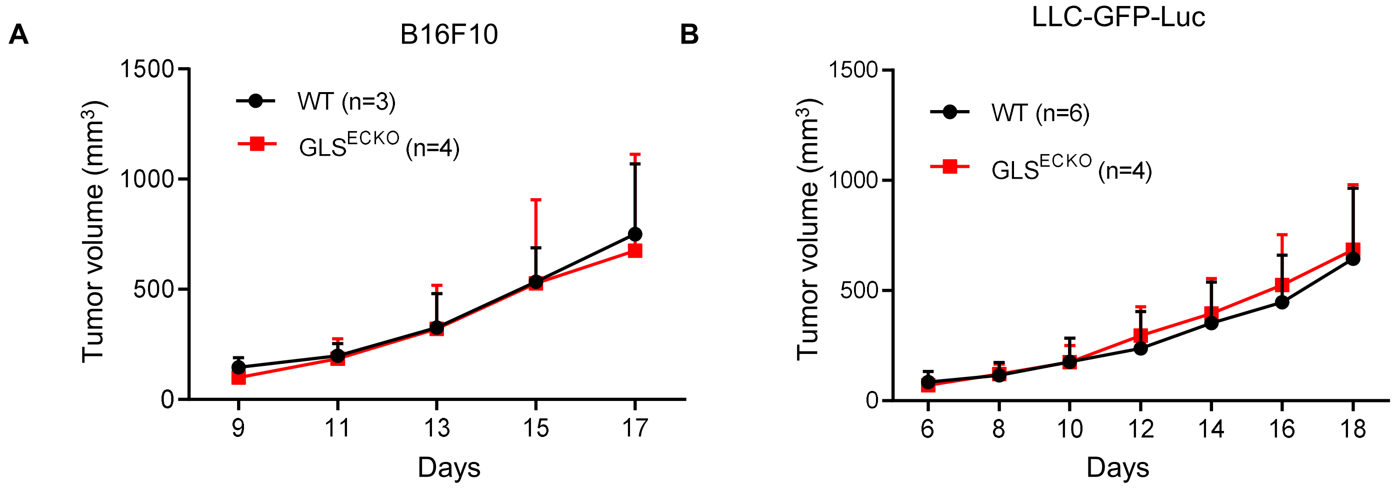

Supplementary Fig. S7: Loss of GLS in the endothelium does not affect subcutaneous tumor growth of (**A**) B16F10 melanoma and (**B**) LLC-GFP-Luc lung cancer models.
