# Supplementary material for: Loss of Vascular Endothelial Glutaminase Inhibits Tumor Growth and Metastasis, and Increases Sensitivity to Chemotherapy
Source: Cancer Res Commun. 2022 Jul 21;2(7):694–705. doi: 10.1158/2767-9764.CRC-22-0048 (PMC9645801; doi:10.1158/2767-9764.CRC-22-0048)
Supplement: Supplementary Materials and Methods1 — This is additional Supplementary Materials and Methods including tables. [file crc-22-0048-s09.pdf]

Supplementary Materials and Methods

Immunohistochemistry: Tumor sections were subjected to immunohistochemistry staining as previously described (1), Briefly tissue sections were fixed in acetone and incubated with 10% H_2O_2 in methanol. The sections were blocked with 2.5% goat serum and incubated at 4°C overnight with leptin antibody (1:100; #AF498-SP, R&D systems). Following several washes, samples were incubated with biotinylated anti-goat IgG antibody (H+L) (BA-9400-1.5, Vector laboratories) for 1 hr at room temperature, followed by streptavidin peroxidase reagents (#51-75477E, BD Pharmingen), liquid diaminobenzidine (DAB) (#00-2014, Invitrogen), and counter stained with hematoxylin. Stained sections were mounted with Cytoseal XYL, and images of at least five fields of view were obtained using an Olympus inverted fluorescence microscope (40×).

RNA Sequencing: RNA was extracted from control (WT) or GLS-deleted ECs (GLS^{ECKO}) using the Direct-zol RNA Miniprep Kit (Zymo Research, Irvine, CA) according to manufacturer's instructions. RNA sequencing was performed by BGI Americas (Cambridge, MA) using the DNBSEQ platform. Raw data was filtered to remove reads with high rates of unknown bases, low quality reads, and reads of adapter sequences. Clean reads were aligned to the reference genome (Mus musculus, version GCF_000001635.26_GRCm38.p6) using HISAT and aligned to reference genes using BowTie2. All analysis was performed using the Dr. Tom platform (BGI). Briefly, differentially expressed genes (DEG) between WT and GLS^{ECKO} endothelial cells were identified using DESeq2 (q value < 0.05), followed by Kyoto Encyclopedia of Genes and Genomes (KEGG) gene enrichment analysis of DEG's. To profile the entire transcriptome in WT and GLS^{ECKO} endothelial cells, gene set enrichment analysis was performed against all KEGG pathways. For both analytical enrichments, all significant pathways (q<0.05) are shown.

Flow cytometry: Tumors were dissociated in RPMI-1640 media (Corning #MT10040CV) supplemented with 5% FBS, 1 mg/ml collagenase IA (Sigma-Aldrich #C9891), and 0.25 mg/ml DNase I (Sigma-Aldrich #DN25) for 30 minutes at 37°C followed by filtering the digested tissue through a 70-μm strainer. Red blood cells were lysed using ACK Lysis Buffer (KD Medical #RGF-3015) and samples washed with PBS before staining with Ghost Dye Violet V510 (Tonbo Biosciences #13-0870) to exclude dead cells. After washing with buffer (0.5% BSA, 2mM EDTA in PBS), samples were blocked in anti- CD16/32 mouse Fc block (Tonbo Biosciences #70-0161) and cell surface proteins were analyzed using antibodies against: CD45, TCRβ, CD4, CD8a, CD25, CD69, CD44, CD62L, and/or CD107a. Intracellular staining for GZMB, IFN-γ, IL-4 and IL-17A, was accomplished using a Cytofix/Cytoperm solution kit (BD, 554714) on paraformaldehyde-fixed cells, according to the manufacturer's protocol. The following markers were used to define cell populations: T cells: $CD45^{+}TCR\beta^{+}$; NK cells: $CD45^{+}TCR\beta^{-}NK1.1^{+}$; Macrophage: $CD45^{+}CD11b^{+}Ly6G^{-}F4/80^{hi}$; M1-like macrophages: $MHCII^{hi}CD206^{-}$; M1-like macrophages: $MHCII^{lo}CD206^{+}$; M-MDSC: $CD45^{+}CD11b^{+}Ly6C^{hi}Ly6G^{-}$; PMN-MDSC: $CD45^{+}CD11b^{+}Ly6C^{-}Ly6G^{hi}$. Fluorescence minus one (FMO) sample were included in the gating controls when needed using splenocytes or tumor cell suspensions. Flow cytometry data was obtained on a BD Fortessa FACSDiva software and analyzed using FlowJo software v10.6.1. Antibodies used in flow panels are detailed in Supplementary table 1.

Supplementary Table 1: Mouse Flow cytometry antibodies

Target	Fluorophore	Source	Dilution
CD4	PE/Dazzle-594	Biolegend #100455	1:500
CD8	VioletFluor 450	BD #560471	1:250
CD11b	FITC	BD # 557396	1:250
CD45	APC-Cy7	BD#557659	1:500
CD107a	PE	BD #558661	1:300
Ly6C	PE-Cy7	eBioscience #25-5932-80	1:1000
Ly6G(Gr1)	PerCP-Cy5.5	Biolegend #127615	1:500
TCR-β	PerCP-Cy5.5	Tonbo #65-5961	1:250
F4/80	APC	eBioscience # 17-4801-82	1:250
Nk1.1	APC	Tonbo #205941-U025	1:200
Granzyme B	PE	Invitrogen #12-8898-82	1:20
IFNγ	APC	BD #554413	1:50
IL-4	FITC	eBioscience #11-7042-82	1:20
MHC Class II (IA/IE)	RedFluro710	Biolegend #107622	1:1000
CD206	PE	Biolegend #141705	1:1000
Ghost	V510	Tonbo #13-0870-T100	1:1000
